# Endothelial Transient Receptor Potential Channels and Vascular Remodeling: Extracellular Ca^2 +^ Entry for Angiogenesis, Arteriogenesis and Vasculogenesis

**DOI:** 10.3389/fphys.2019.01618

**Published:** 2020-01-21

**Authors:** Sharon Negri, Pawan Faris, Roberto Berra-Romani, Germano Guerra, Francesco Moccia

**Affiliations:** ^1^Laboratory of General Physiology, Department of Biology and Biotechnology “L. Spallanzani”, University of Pavia, Pavia, Italy; ^2^Department of Biology, College of Science, Salahaddin University-Erbil, Erbil, Iraq; ^3^Department of Biomedicine, School of Medicine, Benemérita Universidad Autónoma de Puebla, Puebla, Mexico; ^4^Department of Medicine and Health Sciences “V. Tiberio”, University of Molise, Campobasso, Italy

**Keywords:** endothelial cells, endothelial colony forming cells, Ca^2+^ signaling, TRPC, TRPV, TRPM

## Abstract

Vasculogenesis, angiogenesis and arteriogenesis represent three crucial mechanisms involved in the formation and maintenance of the vascular network in embryonal and post-natal life. It has long been known that endothelial Ca^2+^ signals are key players in vascular remodeling; indeed, multiple pro-angiogenic factors, including vascular endothelial growth factor, regulate endothelial cell fate through an increase in intracellular Ca^2+^ concentration. Transient Receptor Potential (TRP) channel consist in a superfamily of non-selective cation channels that are widely expressed within vascular endothelial cells. In addition, TRP channels are present in the two main endothelial progenitor cell (EPC) populations, i.e., myeloid angiogenic cells (MACs) and endothelial colony forming cells (ECFCs). TRP channels are polymodal channels that can assemble in homo- and heteromeric complexes and may be sensitive to both pro-angiogenic cues and subtle changes in local microenvironment. These features render TRP channels the most versatile Ca^2+^ entry pathway in vascular endothelial cells and in EPCs. Herein, we describe how endothelial TRP channels stimulate vascular remodeling by promoting angiogenesis, arteriogenesis and vasculogenesis through the integration of multiple environmental, e.g., extracellular growth factors and chemokines, and intracellular, e.g., reactive oxygen species, a decrease in Mg^2+^ levels, or hypercholesterolemia, stimuli. In addition, we illustrate how endothelial TRP channels induce neovascularization in response to synthetic agonists and small molecule drugs. We focus the attention on TRPC1, TRPC3, TRPC4, TRPC5, TRPC6, TRPV1, TRPV4, TRPM2, TRPM4, TRPM7, TRPA1, that were shown to be involved in angiogenesis, arteriogenesis and vasculogenesis. Finally, we discuss the role of endothelial TRP channels in aberrant tumor vascularization by focusing on TRPC1, TRPC3, TRPV2, TRPV4, TRPM8, and TRPA1. These observations suggest that endothelial TRP channels represent potential therapeutic targets in multiple disorders featured by abnormal vascularization, including cancer, ischemic disorders, retinal degeneration and neurodegeneration.

## Introduction

Blood vessel formation is an obligate requirement during embryonic development to nourish the cells of the rapidly expanding embryo with oxygen (O_2_) and nutrients and to remove their catabolic waste (Fischer et al., 2006; Udan et al., 2013). In addition, the circulatory vasculature serves to stabilize the body temperature and prevent pH unbalance, thereby contributing to maintain the homeostasis of the whole organism (Heinke et al., 2012). Furthermore, the vascular network drives the rapid communication among distant tissues mediated by cytokines and hormones and guides immune cells toward sites of inflammation or infection (Heinke et al., 2012; Udan et al., 2013). Finally, vascular endothelial cells emit instructive, angiocrine signals that regulate resident hematopoietic, epithelial, mesenchymal, cardiac and neuronal cells, as well as their corresponding populations of progenitor and stem cells, to finely orchestrate local metabolism and homeostasis and support organ regeneration independently on blood perfusion (Rafii et al., 2016). Neovessel formation also occurs throughout postnatal life, e.g., in uterine endometrium during the ovarian cycle, embryo implantation and placentation, during skeletal muscle growth and remodeling, during bone morphogenesis, and during wound healing after traumatic injury (Fischer et al., 2006; Chung and Ferrara, 2011). Therefore, insufficient vascularization or impaired perfusion due to vessel obstruction may lead to severe ischemic disorders, including acute myocardial infarction (AMI), stroke, peripheral artery disease (PAD), ischemic retinopathies, pre-eclampsia as well as to neurodegeneration (Fischer et al., 2006; Potente et al., 2011). On the other hand, aberrant vascularization is associated to life-threatening diseases, such as cancer, intraocular and inflammatory diseases, and pulmonary arterial hypertension (PAH) (Chung and Ferrara, 2011).

**FIGURE 1:**
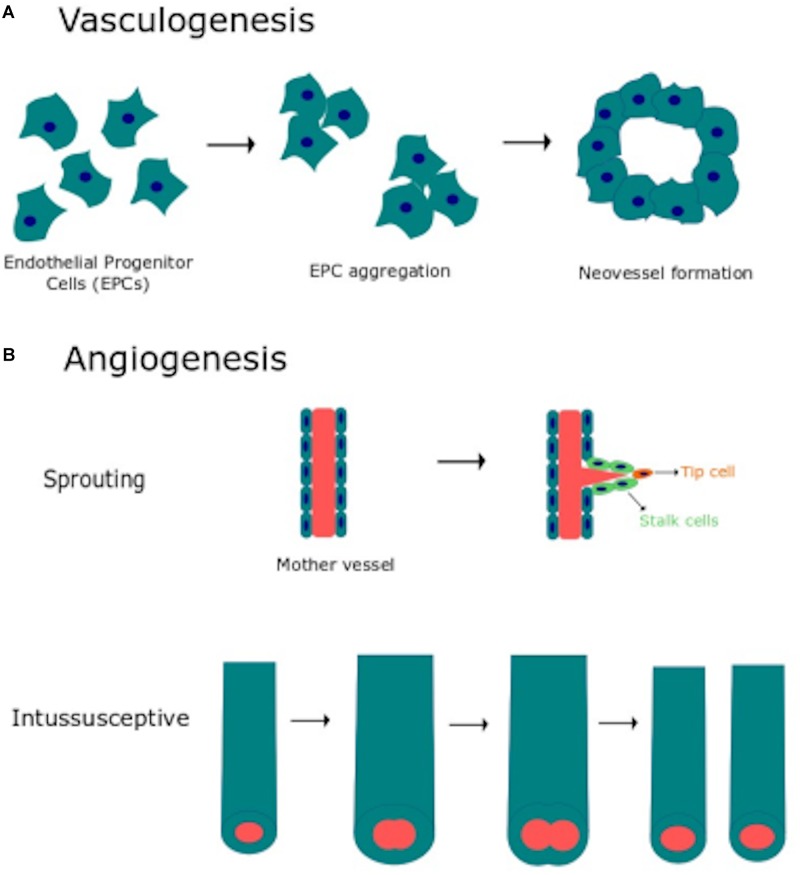
Vasculogenesis and angiogenesis are the main processes responsible for vascular remodeling. **(A)** Schematic representation of vasculogenesis, which consists in *de novo* aggregation of circulating endothelial progenitor cells (EPCs) into functional vessels. **(B)** Schematic representation of angiogenesis, the physiological process whereby capillaries give rise to neovessels to cope with oxygen and nutrient requirements. Angiogenesis may occur through two distinct mechanisms: sprouting angiogenesis and intussusceptive angiogenesis (see text for further details).

Vascular morphogenesis is a multistep process which requires endothelial cell to proliferate, migrate, align in the direction of blood flow, assemble into tubular structures, branch and anastomose with existing vasculature (Chung and Ferrara, 2011; Potente et al., 2011). Two distinct mechanisms, known as vasculogenesis and angiogenesis (Figure 1), are involved in the formation and maintenance of the vascular network both in the developing embryo and during postnatal life (Chung and Ferrara, 2011; Potente et al., 2011). Vasculogenesis consists in the *de novo* aggregation of circulating endothelial progenitor cells (EPCs), also referred to as angioblasts in the developing embryo, into functional vessels (Figure 1A). Subsequent expansion and remodeling of nascent capillary plexus requires the engagement of the angiogenic process, which may be distinguished into sprouting angiogenesis and intussusceptive angiogenesis (Figure 1B) (Fischer et al., 2006; Chung and Ferrara, 2011; Potente et al., 2011). Sprouting angiogenesis is activated when the balance between pro- and anti-angiogenic cues is tipped in favor of pro-angiogenic signals, such as vascular endothelial growth factor (VEGF), basic fibroblast growth factor (bFGF) and platelet derived growth factor (PDGF). Pro-angiogenic stimuli cause an increase in endothelial permeability, which leads to the extravasation of multiple plasma proteins (e.g., fibrinogen and fibronectin) that contribute to establish a provisional scaffold for migrating endothelial cells. Degradation of the basement membrane by matrix metalloproteinases (MMPs) released by the activated endothelium contributes to create the most suitable substrate for endothelial cell migration and to create the space necessary for tubule formation. Accordingly, the endothelial cell closest to VEGF adopts a migratory (non-proliferative) tip cell phenotype, thereby budding from the existing capillary toward the source of the stimulus. Adjacent endothelial cells experience lower VEGF levels, adopt a proliferative (non-migrating) stalk phenotype and trail behind the tip cell, thereby elongating the vessel sprout. Thereafter, the neovessel sprout comes in contact and fuses either with a neighboring angiogenic sprout or with a functional capillary, through a process known as anastomosis, which may involve, respectively, two or one tip cells. Therefore, sprouting endothelial cells assemble into a multicellular tube, which undergoes a complex remodeling leading to lumen formation, connection between parental vessels and functional blood flow (Fischer et al., 2006; Chung and Ferrara, 2011; Potente et al., 2011). Subsequently, naked endothelial cells become quiescent by adopting the “cobblestone”-like phalanx phenotype and the nascent vessel is further stabilized by the PDGF-dependent recruitment of mural cells, such as pericytes and vascular smooth muscle cells (VSMCs) (Potente et al., 2011). Microvascular growth may also be accomplished by intussusceptive angiogenesis, which consists in the insertion of a transcapillary pillar followed by the expansion of pillar diameter and consequent splitting of the existing capillary (Udan et al., 2013). It is now clear that EPCs play a crucial role in maintaining endothelial homeostasis and in restoring local blood perfusion upon an ischemic insult also in the adults (D’Alessio et al., 2015; Banno and Yoder, 2018). In addition, EPCs may be mobilized in peripheral circulation to sustain the angiogenic switch during the early phases of tumor growth (Moccia et al., 2015; Poletto et al., 2018). Finally, ischemic neovascularization may also impinge on arteriogenesis, including collateralization, which denotes the growth and remodeling of existing arterioles into larger vessels when a main artery is occluded (Heil et al., 2006).

It has long been known that endothelial Ca^2+^ signals play a crucial role in angiogenesis and arteriogenesis (Simons et al., 2016). Multiple pro-angiogenic cues, such as VEGF, bFGF, PDGF, epidermal growth factor (EGF), stromal derived factor-1α (SDF-1α), and angiopoietins regulate endothelial cell behavior and fate through an increase in intracellular Ca^2+^ concentration ([Ca^2+^]_i_) (Moccia et al., 2003; Yokota et al., 2015; Noren et al., 2016; Savage et al., 2019). Likewise, VEGF and SDF-1α induce pro-angiogenic Ca^2+^ signals in endothelial colony forming cells (ECFCs) (Dragoni et al., 2011, 2013; Zuccolo et al., 2018; Moccia, 2020), which represent the only EPC subtype truly belonging to the vascular endothelial lineage (Medina et al., 2017; Poletto et al., 2018). Transient Receptor Potential (TRP) channels regulate numerous endothelial cell functions by mediating extracellular Ca^2+^ entry in response to chemical, mechanical and thermal stimuli (Moccia et al., 2012a; Earley and Brayden, 2015; Guerra et al., 2018; Smani et al., 2018; Thakore and Earley, 2019). Herein, we describe how endothelial TRP channels stimulate vascular remodeling by promoting angiogenesis, arteriogenesis and vasculogenesis through the integration of multiple environmental and intracellular cues. We then describe how remodeling of the endothelial blend of TRP channels could promote aberrant vascularization in solid tumors. Understanding how TRP channels regulate endothelial cell fate is indispensable to properly target them to rescue blood perfusion in ischemic disorders and interfere with the vascular network in i.e., cancer and intraocular disorders.

## TRP Channels: an Introduction

The TRP channel superfamily of non-selective cation channels comprises 28 members that are subdivided in six subfamilies by sequence homology: TRP Canonical (TRPC1-7), TRP Vanilloid (TRPV1-6), TRP Melastatin (TRPM1-8), TRP Ankyrin 1 (TRPA1), TRP Mucolipin (TRPM1-3), and TRP Polycystin (TRPP) (Gees et al., 2010; Earley and Brayden, 2015; Smani et al., 2018; Thakore and Earley, 2019). The TRPP subfamily consists of eight members, although only TRPP2, TRPP3 and TRPP5 display the molecular architecture and function of an ion channel (Gees et al., 2010). Moreover, TRPC2, which plays a crucial role in acrosomal reaction and pheromone detection in mice, is only a pseudo-gene in humans (Gees et al., 2010). Herein, we briefly survey the basic features of TRP channel structure and illustrate their main electrophysiological properties before focusing on the pro-angiogenic role of endothelial TRP channels.

### The Topological Organization of TRP Channels

The molecular topology of TRP channels comprises six transmembrane (TM1-6) α-helix segments, a reentrant pore loop between TM5 and TM6, and cytosolic NH_2_- and COOH-terminal tails. TRP channel subunits assemble into a tetrameric complex around a central cation conducting pathway lined by TM5, TM6 and their interconnecting pore loop (Gaudet, 2008). This structure is highly reminiscent of that described in voltage-gated K^+^ channels, although TRP channels lack a voltage-sensor in TM4 (Gaudet, 2008). The NH_2_- and COOH-terminal tails flanking the TM domain may greatly differ in length between different TRP subfamilies and may fulfill diverse functions, such as engaging subunit interactions and providing regulatory domains for cytosolic kinases and other cellular/cytoskeletal proteins as well as serving as binding sites for Ca^2+^ and Ca^2+^-dependent sensors, including calmodulin (CaM) and STIM1 (Gaudet, 2008; Birnbaumer, 2009; Gees et al., 2010). For instance, a 25 amino acid conserved TRP domain is located immediately distal to TM6 in TRPC, TRPM and TRPV members, while it is absent in other TRP subfamilies (Birnbaumer, 2009). The TRP domain includes two highly conserved sequences, known as TRP-box 1 and TRP-box 2, bordering a central region, which displays a higher degree of sequence variability (Venkatachalam and Montell, 2007). TRP-box 1 consists of three variations of the Glu-Trp-Lys-Phe-Ala-Arg (EWKFAR) motif and provides the binding site for phosphatidylinositol phosphates, such as phosphatidylinositol-4,5-bisphosphate (PIP_2_) (Birnbaumer, 2009), or is involved in subunit assembly (Venkatachalam and Montell, 2007). TRP-box 2 is a prolin-rich sequence, which is variable between different TRPC and TRPM subunits, while it is absent in TRPV channels (Venkatachalam and Montell, 2007). In addition, TRPC, TRPV and TRPA1 channels share multiple NH_2_-terminal ankyrin repeat domains (ARDs), which fulfill numerous functions, including cytoskeleton interaction and channel opening by membrane deformation (“spring hypothesis”), channel gating upon covalent modifications induced by extracellular ligands, and channel tetramerization (Gaudet, 2008). While TRPCs and TRPVs contain 3-4 ARDs, TRPA1 displays up to 14-19 ARDs at the NH_2_-terminal tail (Gees et al., 2010). Coiled coil domains, which are predicted to mediate subunit assembly and channel function, have been detected at the NH_2_- and COOH-termini of TRPCs, TRPVs, TRPMs, and TRPPs (Li M. et al., 2011). Moreover, coiled coil domains support the physical association between TRPC channels and the endoplasmic reticulum (ER) Ca^2+^ sensor, STIM1 (Lee et al., 2014). Intriguingly, the COOH-terminal tail of TRPM2, TRPM6 and TRPM7 displays functional enzymatic domains that may be involved in channel gating and catalyze specific cellular reactions. TRPM2 is featured by a NUDIX phosphohydrolase domain, which binds to and hydrolyses adenosine diphosphate ribose-ribose (ADPr) into 5-phosphate and adenosine monophosphate (AMP); ADPr, in turn, gates TRPM2, whereas AMP negatively modulates channel gating (Faouzi and Penner, 2014). TRPM6 and TRPM7 show an α-kinase domain, which consists in an atypical serine-threonine protein kinase targeting multiple targets, including myosin IIA, IIB, and IIC, elongation factor 2 kinase, annexin A1 (Fleig and Chubanov, 2014). Moreover, this COOH-terminal kinase may be proteolytically cleaved in a cell-specific manner, thereby translocating into the nucleus and regulating chromatin organization and gene expression through histone phosphorylation (Krapivinsky et al., 2014).

### TRP Subunits Assemble Into Homomeric or Heteromeric Channels

All TRP subunits may preferentially aggregate into homomeric cation channels (Lepage and Boulay, 2007), although a recent investigation failed to detect functional TRPC1 tetramers in a heterologous expression system (Storch et al., 2012). However, TRP channels may also assemble into heteromeric channels by combining with subunits belonging to either the same subfamily or different subfamilies (Ma et al., 2011a; Berrout et al., 2012; Storch et al., 2012). Heteromeric TRP channels have been widely reported in naïve cells, including vascular endothelial cells, and heterologous expression systems and present biophysical fingerprints and regulatory mechanisms distinct from their homomeric counterparts (Cheng et al., 2010). Subunit heteromerization has been extensively investigated in the TRPC subfamily. For instance, TRPC1 may assemble into functional heteromeric channels with TRPC3, TRPC4, or TRPC5 (Smani et al., 2018), while TRPC3, TRPC6, and TRPC7 may assemble into functional heteromeric channels in both naïve tissues (Goel et al., 2002) and heterologous expression systems (Hofmann et al., 2002). TRPV subunits may also associate into heterotetramers. For instance, any two members of TRPV1-4 subunits assembly into functional heteromeric channels that are exclusively located on the plasma membrane (Cheng et al., 2007), as well as several investigations demonstrated that TRPV5/TRPV6 subunits assemble into heteromeric channel complexes (Cheng et al., 2010). Furthermore, functional heterotetramers comprising TRP channel subunits from different subfamilies have widely been reported and include the following combinations: TRPC1/TRPP2 (Berrout et al., 2012), TRPC1/TRPV4 (Ma et al., 2011a), TRPC1/TRPV6 (Schindl et al., 2012), TRPV4/TRPC6 (Goldenberg et al., 2015), and TRPML3/TRPV5 (Guo et al., 2013).

### Gating Mechanisms of TRP Channels

TRP channels are polymodal cellular sensors that may be gated by a plethora of chemical and physical stimuli, including intracellular second messengers [e.g., diacylglycerol (DAG), arachidonic acid (AA), ADPr and hydrogen peroxide (H_2_O_2_)], intracellular ions (e.g., an increase in cytosolic H^+^ and Ca^2+^ and a decrease in cytosolic Mg^2+^), dietary agonists (e.g., capsaicin, menthol, and allylisothiocyanate or AITC), synthetic ligands (e.g., 4α-phorbol-12,13-didecanoate), gasotransmitters [e.g., nitric oxide (NO)], proteins (e.g., G proteins), mechanical perturbation (e.g., membrane stretch, osmotic swelling and laminar shear stress), and temperature fluctuations (Venkatachalam and Montell, 2007; Gees et al., 2010; Earley and Brayden, 2015; Guerra et al., 2018; Samanta et al., 2018; Smani et al., 2018; Thakore and Earley, 2019). In addition, some members of the TRPC subfamily may be recruited by depletion of the ER Ca^2+^ store via direct binding with STIM1 and/or Orai1 or with inositol-1,4,5-trisphosphate (InsP_3_) receptors (InsP_3_Rs) (Xi et al., 2008; Di Buduo et al., 2014; Lee et al., 2014; Ambudkar et al., 2017).

### Biophysical Properties of TRP Channels

TRP channels are permeable to monovalent (i.e., Na^+^ and K^+^) and divalent (e.g., Ca^2+^ and Mg^2+^) cations, although their relative permeability to Ca^2+^ and Na^+^ (P_Ca_/P_Na_) may widely change among the different subunits. For instance, TRPV5 and TRPV6 display the largest P_Ca_/P_Na_ (>100), while TRPM4 and TRPM5 are virtually impermeable to Ca^2+^ (P_Ca_/P_Na_ < 0.01), although they are gated by an increase in [Ca^2+^]_i_ (Gees et al., 2010). The P_Ca_/P_Na_ of the remaining TRP channels is situated within these extremes of the selectivity spectrum, ranging from 0.5 (TRPM2) up to 20 (TRPV1) (Gees et al., 2010). Nevertheless, TRPM6 and TRPM7 are more permeable to Mg^2+^, whereas TRPV1, TRPML1, and TRPP3 display a high permeability to H^+^. The resting membrane potential (V_M_) of vascular endothelial cells, which may scatter between −88 and −20 mV (Voets et al., 1996; Moccia et al., 2002), is more negative than the reversal potential for Na^+^ and Ca^2+^ (E_Na_ = E_Ca_ ≈ +60 mV), but more positive than the reversal potential for K^+^ (E_K_ = −88 mV). Therefore, in response to extracellular stimulation, TRP channels conduct inward Na^+^ (and Ca^2+^ or Mg^2+^) currents, which exert a profound impact on intracellular Ca^2+^ dynamics. Accordingly, Ca^2+^ entry through Ca^2+^-permeable TRP channels may directly increase the [Ca^2+^]_i_, whereas Na^+^ influx induces membrane depolarization, thereby activating voltage-gated Ca^2+^ channels in excitable cells and modulating the driving-force for Ca^2+^ entry in non-excitable cells (Gees et al., 2010; Curcic et al., 2019). The functional impact exerted by Ca^2+^-permeable TRP channels on intracellular Ca^2+^ homeostasis depends, therefore, on their “fractional Ca^2+^ current,” that has not been estimated for all TRP channels. For instance, apart from TRPV5 and TRPV6 that mediate real Ca^2+^ currents, TRPA1 and TRPM3 exhibit fractional Ca^2+^ currents of ∼20%, while TRPV1 shows a fractional Ca^2+^ current of ∼5% (Gees et al., 2010; Earley and Brayden, 2015). The capability of TRP channels to generate a substantial increase in [Ca^2+^]_i_ has actually been fully appreciated in vascular endothelial cells loaded with the Ca^2+^-sensitive fluorophore, Fluo-3 or Fura-2, or expressing genetically encoded Ca^2+^ indicators, such as GCaMP, by using high-speed confocal and total internal reflection (TIRF) microscopy (Earley and Brayden, 2015; Thakore and Earley, 2019). A recent series of investigations characterized the Ca^2+^ entry events through unitary or clusters of functionally coupled endothelial TRP channels, also known as TRP Ca^2+^ sparklets. Ca^2+^ sparklets were measured from both primary endothelial cells and intact endothelium upon stimulation of TRP channels displaying sufficient Ca^2+^ permeability and unitary conductance (Sonkusare et al., 2012; Pires et al., 2015; Sullivan et al., 2015), such as TRPV1, TRPV4, and TRPA1. It has been estimated that the amount of extracellular Ca^2+^ entering vascular endothelial cells through individual TRPV4 Ca^2+^ sparklets exceeds by ∼100 times that recorded during voltage-gated L-type Ca^2+^ sparklets. Of note, the unitary amplitudes of TRPA1 and TRPV3 Ca^2+^ sparklets are, respectively, two- and threefold greater that those recorded for TRPV4 Ca^2+^ sparklets (Sonkusare et al., 2012; Pires et al., 2015; Sullivan et al., 2015). This finding confirms that some TRP channels are able to generate a large increase in local Ca^2+^ concentration, which could be remain spatially confined to the submembranal space or propagate to the bulk cytosol via the Ca^2+^-dependent recruitment of InsP_3_Rs and ryanodine receptors (RyRs) (Earley and Brayden, 2015; Thakore and Earley, 2019).

## TRP Channels in Endothelial Cells

Endothelial cells line the inner lumen of blood vessels and are, therefore, exposed to a myriad of chemical (e.g., growth factors and chemokines) and physical (e.g., laminar shear stress and pulsatile stretch) cues, which must be properly perceived for the maintenance of tissue homeostasis (Moccia et al., 2012a,2019b). TRP channels represent the most suitable signal transduction pathway whereby vascular endothelial cells integrate such a vast array of extracellular stimuli due to the versatility of their gating mechanisms. Accordingly, endothelial TRP channels serve as polymodal cellular sensors as, with the exception of TRPC3, TRPC6 and TRPM4, they are sensitive to multiple signaling pathways (Earley and Brayden, 2015; Smani et al., 2018; Thakore and Earley, 2019).

### Endothelial TRP Channels Expression

Most mammalian TRP isoforms were reported in vascular endothelial cells, including: TRPC1-7; TRPV1-4; TRPA1; TRPP1-2; and TRPM1-8 except TRPM5 (Moccia et al., 2012a; Earley and Brayden, 2015; Smani et al., 2018; Thakore and Earley, 2019). However, the pattern of TRP channel distribution may vary across the vascular tree and in different animal species. For instance, TRPC3 is widely expressed in human, but not bovine, pulmonary artery endothelial cells, whereas TRPC1 is present in mouse, but not rat, aortic endothelial cells (Moccia et al., 2012a). Finally, mouse, but not human, brain microvascular endothelial cells selectively express TRPC1-6 channels (Brown et al., 2008; Zuccolo et al., 2019). Nevertheless, conflicting reports about the expression of specific TRP isoforms in the same endothelial cell type suggest that cell culture conditions and expression detection techniques may affect our knowledge of endothelial TRP channel distribution. Notably, the blend of ion channels endowed to endothelial cells may be remarkably different *in vitro* and may be further affected by serial passages of cultured cells (Moccia et al., 2012a; Earley and Brayden, 2015; Smani et al., 2018; Thakore and Earley, 2019). It is, therefore, mandatory that the expression of each TRP isoform along the vascular tree is confirmed *in vivo*. As reported in other cell types, naïve TRP channels may consist of heteromeric subunits also in vascular endothelial cells. The following endothelial TRP channel complexes were reported: TRPC1-TRPC4 (Cioffi et al., 2012; Sundivakkam et al., 2012; Greenberg et al., 2017), TRPC1-TRPV4 (Ma et al., 2011a, b), TRPC3-TRPC4 (Poteser et al., 2006), TRPV1-TRPV4 (O’Leary et al., 2019), TRPP2-TRPC1 (Berrout et al., 2012), and TRPC1-TRPP2-TRPV4 (Du et al., 2014). As anticipated earlier, heteromerization expands the range of gating properties and biophysical properties of endothelial TRP channels, thereby boosting their impact on the regulation of endothelial cell functions.

### TRP Channels Modulate the [Ca^2+^]_i_ and V_M_ in Endothelial Cells

TRP channels regulate virtually all endothelial cell functions by generating either a spatially restricted Ca^2+^ domain around the cytosolic mouth of the channel pore or a global increase in [Ca^2+^]_i_ (Curcic et al., 2019; Thakore and Earley, 2019). TRP channels mediate Ca^2+^ entry in vascular endothelial cells exposed to a myriad of autacoids and hormones, including acetylcholine, ATP, bradykinin, erythropoietin, histamine, and angiotensin II, or to mechanical stimuli, such as pulsatile stretch and laminar shear stress (Moccia et al., 2012a; Earley and Brayden, 2015; Smani et al., 2018; Thakore and Earley, 2019). In addition, TRP channels modulate endothelial V_M_ by conducting a depolarizing inward current, carried by Na^+^ and Ca^2+^ (with the exception of the Ca^2+^-impermeable TRPM4), thereby causing a positive shift in V_M_ that could be boosted by the Ca^2+^-dependent recruitment of TRPM4 (yet to be demonstrated) (Moccia et al., 2012a; Earley and Brayden, 2015; Smani et al., 2018; Thakore and Earley, 2019). Furthermore, TRP channels-induced membrane depolarization could activate endothelial voltage-gated channels, although this issue remains highly controversial (Moccia et al., 2012a). Notably, human umbilical vein endothelial cells (HUVECs) may express NaV1.5 and NaV1.7, which represent the pore-forming α-subunits of, respectively, cardiac and neuronal voltage-gated Na^+^ channels (Andrikopoulos et al., 2011b). Likewise, CaV3.1 T-type voltage-gated Ca^2+^ channels are expressed in pulmonary microvascular endothelial cells (Zheng et al., 2019) as well as L- and R-type voltage-gated Ca^2+^ current were recorded in several types of endothelial cells (Moccia et al., 2012a). Alternately, when coupled to the Ca^2+^-activated intermediate- and small-conductance K^+^ channels (IK_Ca_ and SK_Ca_, respectively), TRP channels may result in endothelial-dependent hyperpolarization (EDH). EDH may in turn be electrotonically propagated to adjacent VSMCs, thereby promoting vasodilation and increasing local blood flow in mesenteric arteries and cerebral microcirculation (Earley and Brayden, 2015; Guerra et al., 2018; Moccia et al., 2019a; Thakore and Earley, 2019).

### TRP Channels Regulate Multiple Endothelial Cell Functions

We refer the reader to a number of recent reviews that provide a comprehensive description of the functional role played by endothelial TRP channels (Earley and Brayden, 2015; Smani et al., 2018; Thakore and Earley, 2019). Herein, we briefly recall that endothelial TRP channels may either mediate short-term responses, e.g., vasodilation or increase in vascular permeability, or support long-lasting processes, e.g., gene expression, proliferation and migration. For instance, acetylcholine recruits TRPC4 to induce NO release and vasodilation in mouse aortic endothelial cells (Freichel et al., 2001), whereas it impinges on TRPV4 to trigger NO- and EDH-dependent vasorelaxation in mouse small mesenteric arteries (Zhang et al., 2009). Likewise, direct activation of TRPV4 with the specific agonist GSK1016790A (GSK) induces vasodilation in rat pulmonary artery by promoting NO release and EDH activation (Addison et al., 2016). Moreover, dietary manipulation may result in robust vasodilation, thereby increasing local blood flow, in several vascular beds. For instance, the plant-derived flavone apigenin stimulates TRPV4 to induce EDH-dependent vasodilation in rat mesenteric arteries (Ma et al., 2012), whereas carvacrol, a pungent ingredient of oregano, and AITC, which is abundant in mustard oil, recruit the EDH pathway by, respectively, stimulating TRPV3 (Pires et al., 2015) and TRPA1 (Earley et al., 2009) in mouse cerebrovascular endothelial cells. Finally, a heteromeric channel formed by TRPC1, TRPV4 and TRPP2 mediates flow-induced vasodilation in mesenteric arteries (Du et al., 2014), whereas laminar shear stress activates the TRPP1-TRPP2 complex to induce NO release and vasodilation in mouse aorta (AbouAlaiwi et al., 2009). Multiple TRP channels, including TRPC1, TRPC3, TRPC4, TRPC6, TRPV4, TRPM2, and TRPM4, may also modulate vascular permeability (Earley and Brayden, 2015; Thakore and Earley, 2019). For instance, TRPC1 and TRPC4 mediate thrombin-induced permeability increase in HUVEC monolayers (Paria et al., 2006) and mouse lung vasculature (Tiruppathi et al., 2002), respectively. Likewise, TRPC3 overexpression/hyperactivation has been associated to the exaggerated increase in blood-brain barrier (BBB) permeability observed in response to status epilepticus (Ryu et al., 2013). Furthermore, TRPM2 mediates H_2_O_2_-induced endothelial hyperpermeability in human pulmonary artery endothelial cells (Hecquet et al., 2008), whereas TRPC6 sustains endotoxin-induced disruption of pulmonary vascular barrier (Tauseef et al., 2012) and pulmonary ischemia-reperfusion evoked edema (Weissmann et al., 2012) in mice. Endothelial TRPM4 is up-regulated in a rodent model of spinal cord injury, thereby resulting in capillary fragmentation with secondary hemorrhage, which further exacerbates the damage to surrounding tissues (Gerzanich et al., 2009). Finally, systemic activation of endothelial TRPV4 with GSK is responsible for the dramatic increase in microvascular permeability and hemorrhage reported in the lung, intestine, and kidney (Willette et al., 2008). In agreement with this observation, lung injury induced by high vascular pressure is mediated by TRPV4-mediated Ca^2+^ entry in capillary endothelial cells (Jian et al., 2008). Extracellular Ca^2+^ entry through TRP channels may also lead to the recruitment of a number of Ca^2+^-dependent transcription factors, including nuclear factor-kappaB (NF-κB) (Thippegowda et al., 2010), activating protein-1 (AP-1; i.e., c-Fos and c-Jun) (Fantozzi et al., 2003), and nuclear factor of activated T cells (NFAT) (Zhu et al., 2019). Endothelial TRP channels could also serve as sensors of oxidative stress and local temperature changes. For instance, H_2_O_2_ induces an aberrant increase in [Ca^2+^]_i_ by activating TRPM2 in pulmonary artery endothelial cells and after ischemia-reperfusion injury in mouse brain microcirculation (Hecquet et al., 2008; Pires and Earley, 2017). Likewise, oxidative stress is detected by a heteromeric channel formed by TRPC3 and TRPC4 in porcine aortic endothelial cells (Poteser et al., 2006), and by TRPA1 (Sullivan et al., 2015) and TRPV3 (Yoshida et al., 2006) in mouse cerebrovascular endothelial cells, while it is unclear whether TRPC5 and TRPV4 are sensitive to redox signaling (Pires and Earley, 2017). It is worth noting that a recent report suggested that redox regulation of the endothelial TRPA1 under hypoxic conditions attenuates ischemic damage following brain stroke by inducing cerebral artery relaxation (Pires and Earley, 2018). Finally, TRPV4 is able to detect a moderate increase in temperature (from 19 to 38°C) in mouse aortic endothelial cells (Watanabe et al., 2002), whereas TRPV1 mediates Ca^2+^ entry when heating from room temperature to over 40°C (Mergler et al., 2010). This sensitivity has been related to temperature-dependent changes in NO release, i.e., in vascular tone, and in endothelial permeability (Watanabe et al., 2002; Mergler et al., 2010). Besides endothelial cells, within the vascular wall, TRP channels are also largely expressed in VSMCs, thereby regulating multiple functions, including proliferation, migration, contractility, and mechanosensation (Inoue et al., 2006; Earley and Brayden, 2015; Randhawa and Jaggi, 2015).

## Endothelial TRP Channels Regulate Angiogenesis and Arteriogenesis

Endothelial TRP channels have long been associated to angiogenesis and vascular remodeling due to their ability to engage intracellular signaling pathways associated to endothelial cell proliferation, migration, adhesion, tubulogenesis, and permeability (Moore et al., 1998; Fantozzi et al., 2003; Cheng et al., 2006). Endothelial TRP channels may control angiogenesis by mediating Ca^2+^ entry in response to extracellular growth factors, such as VEGF and bFGF, that are liberated in peripheral circulation upon an ischemic insult or by growing tumors (Moccia et al., 2019b). Alternately, TRP channels sense cellular stress factors, such as an increase in ROS production or a decrease in Mg^2+^ levels, or an increase in laminar shear stress, which ultimately stimulates arteriogenesis (Moccia and Guerra, 2016). Finally, endothelial TRP channels could promote vascular remodeling following stimulation with synthetic agonists.

### The Role of TRPC in Angiogenesis

Endothelial TRPC channels sustain angiogenesis by sustaining the Ca^2+^ response to pro-angiogenic cues, including VEGF, bFGF, and insulin-like growth factor 1 (IGF-1), and/or by detecting subtle changes in the composition of the local microenvironment, and/or in response to small molecule drugs (Smani et al., 2018; Moccia et al., 2019b). While the mechanisms whereby TRPC3, TRPC4, TRPC5 and TRPC6 control angiogenesis has been largely understood, the role of TRPC1 is still shrouded in mystery.

#### General Mechanisms for Growth Factors-Induced Intracellular Ca^2+^ Signals in Endothelial Cells

An increase in endothelial [Ca^2+^]_i_ is indispensable to sustain the pro-angiogenic effect of growth factors and chemokines (Moccia et al., 2012b, 2019b; Smani et al., 2018). Growth factors bind to their cognate receptor tyrosine kinase (RTKs), thereby stimulating phospholipase C-γ1 (PLCγ1) to cleave phosphatidylinositol 4,5-bisphosphate (PIP_2_), a minor phospholipid component of the plasma membrane, into the intracellular second messengers, InsP_3_ and DAG (Moccia et al., 2019b). InsP_3_, in turn, triggers endogenous Ca^2+^ release by gating InsP_3_Rs, which are Ca^2+^-permeable, non-selective cation channels embedded in ER membrane (Moccia et al., 2019b). VEGF-induced InsP_3_-dependent ER Ca^2+^ release may require the concomitant mobilization of endo-lysosomal Ca^2+^ through two-pore channel 2 (TPC2) (Favia et al., 2014), while it is barely supported by RyRs, as recently demonstrated in human aortic endothelial cells (HAECs) (Evangelista et al., 2012). InsP_3_-induced intracellular Ca^2+^ release results in a dramatic fall in ER Ca^2+^ concentration ([Ca^2+^]_ER_) that is detected by STIM1, a single-pass transmembrane protein that senses [Ca^2+^]_ER_ (Moccia et al., 2012b). Once activated, STIM1 oligomerizes and translocates toward peripheral ER-plasma membrane junctions (≈ 20 nm), known as *puncta*, where it tethers and gates Orai1, the pore-forming subunits of the Ca^2+^ release-activated Ca^2+^ (CRAC) channels (Moccia et al., 2019b), thereby inducing store-operated Ca^2+^ entry (SOCE). The pro-angiogenic role of SOCE has been mainly evaluated upon endothelial cell stimulation with VEGF, the master regulator of vascular remodeling (Moccia et al., 2019b). The physical association between STIM1 and Orai1 mediate VEGF-induced SOCE, proliferation and *in vitro* tubulogenesis in HUVECs (Abdullaev et al., 2008; Li J. et al., 2011) and HAECs (Evangelista et al., 2012). Of note, Li J. et al. (2011) failed to detect VEGF-evoked inward Ca^2+^ currents in HUVECs, which is consistent with the notion that the CRAC current (I_CRAC_) may fall below the resolving power of whole-cell patch clamp recording systems.

#### The Controversial Role of TRPC1 and TRPC4 in VEGF-Induced Extracellular Ca^2+^ Entry

Nevertheless, earlier work suggested that TRPC1 contributed to VEGF-induced SOCE and store-operated Ca^2+^ current (known as I_SOC_) in HUVECs (Jho et al., 2005). The I_SOC_ displays strikingly distinct biophysical features as compared to I_CRAC_, including a linear current-to-voltage relationship, a reversal potential (E_rev_) approximately equal to 0 mV, and a single-channel conductance in the pS range (Moccia et al., 2019b). Accordingly, Jho et al. (2005) detected a VEGF-induced inward Ca^2+^-permeable current, which clearly resembled the TRPC1-mediated current previously described in VSMCs with a contractile phenotype (Shi et al., 2017) and in human salivary gland cells (Cheng et al., 2011). Moreover, a recent report confirmed that VEGF-induced SOCE in HUVECs was affected by genetic silencing of TRPC1 (Kusaba et al., 2010). Notably, Abdullaev et al. (2008) also reported that genetic deletion of TRPC1 (and TRPC4) did not affect the I_CRAC_ development and amplitude, but retarded cell growth in HUVECs. Several hypotheses have been put forward to explain this puzzling controversy. For instance, the intracellular recording solution employed in Abdullaev et al. (2008) and Li J. et al. (2011) has been widely employed to record the I_CRAC_ rather than the I_SOC_ (Cs^+^, which is poorly permeable through Orai1 channels, as main intracellular cation). Notably, Jho et al. (2005) reported that VEGF-induced I_SOC_ was sensitive to 1 μM La^3+^, which selectively targets Orai1 rather than TRPC1 (Prakriya and Lewis, 2015). In addition, STIM1 may also interact with TRPC1 and TRPC4 in vascular endothelial cells (Ma et al., 2011a; Sundivakkam et al., 2012). Therefore, one could speculate that Orai1-mediated extracellular Ca^2+^ entry triggers TRPC1 insertion into the plasma membrane, followed by STIM1-dependent recruitment, as demonstrated in human salivary gland cells (Cheng et al., 2011). Alternately, it has been shown that the endothelial Orai1 may assemble and determine the Ca^2+^-selectivity of a heteromeric complex also involving TRPC1, TRPC4 and, possibly, TRPV4 (Brough et al., 2001; Cioffi et al., 2005; Ma et al., 2011a; Cioffi et al., 2012; Xu et al., 2015). Therefore, the controversial contribution of TRPC1 (and TRPC4) to VEGF-induced endothelial SOCE may reflect multiple factors, including cell source, cell passage, culture medium, substrate of adhesion, experimental protocols, detection methods and so on (Beech, 2012). We refer the reader to a number of excellent review articles dealing with the ongoing controversy regarding the molecular make-up of endothelial SOCE (Blatter, 2017; Groschner et al., 2017).

#### TRPC1 and TRPC4 in Angiogenesis

**FIGURE 2:**
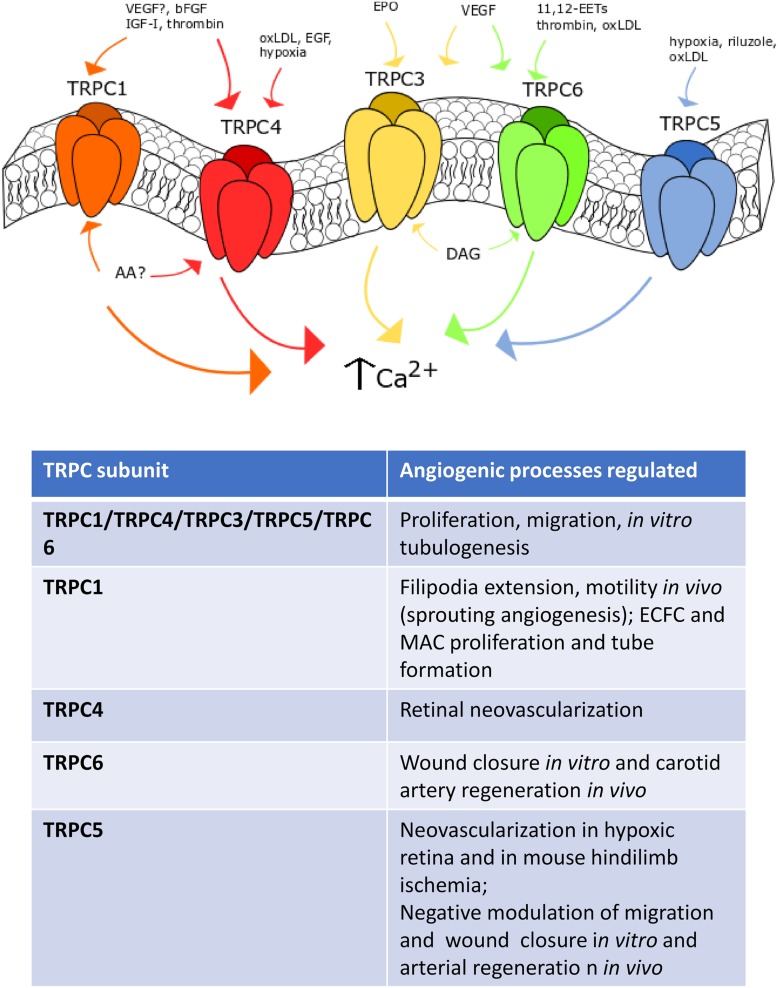
TRPC channels in angiogenesis. Representation of the main TRPC channels involved in angiogenesis. TRPC1 mediates angiogenesis after VEGF- (still debated), IGF-, bFGF- and thrombin-induced stimulation. TRPC4 is sensitive to EGF, oxLDL and hypoxia. TRPC3 responds to erythropoietin (EPO) and VEGF stimulation, such as TRPC6, which can be also activated by oxLDL, thrombin and 14,15-EETs. Finally, TRPC5 is sensitive to hypoxia, riluzole and oxLDL-dependent activation.

Whatever the gating mechanism, TRPC1 and TRPC4 do play a role in angiogenesis, as more widely illustrated in the next paragraphs (Figure 2).

##### TRPC1

As recently reviewed in Moccia et al. (2019b), bFGF evokes extracellular Ca^2+^ entry in bovine aortic endothelial cells (BAECs) by binding to FGF receptor-1 (FGFR-1) and stimulating cytosolic phospholipase A2, phospholipase D (PLD) and PLC to release AA, which in turn recruits a heteromeric TRPC1/TRPC4 channel (Antoniotti et al., 2002, 2003, 2006). Blocking AA production and preventing Ca^2+^ influx with carboxyamidotriazole (CAI) inhibit BAEC proliferation (Antoniotti et al., 2003), thereby suggesting that the heteromeric TRPC1/TRPC4 channel underlies the pro-angiogenic effect of bFGF. Interestingly, IGF-I elicits extracellular Ca^2+^ influx by engaging the same Ca^2+^-permeable route as that stimulated by bFGF in BAECs (Munaron and Fiorio Pla, 2000). In agreement with these observations, genetic silencing of TRPC1 by means of antisense morpholino oligonucleotides prevent filopodia extension, motility and proliferation in endothelial tip cells of zebrafish larvae (Yu et al., 2010). TRPC1 was found to drive sprouting angiogenesis by mediating VEGF-induced extracellular signal-regulated kinases (ERK) in intersegmental vessels (Yu et al., 2010). Endothelial Ca^2+^ oscillations sustain VEGF-induced filopodia elongation and endothelial cell migration during angiogenesis in zebrafish (Savage et al., 2019), but the role of TRPC1 in this signaling remains to be elucidated. While these studies provide the evidence that TRPC1 sustains angiogenesis, other reports argue against this conclusion. For instance, genetic silencing of TRPC1 did not affect the spontaneous Ca^2+^ transients that drive *in vitro* tubulogenesis in EA.hy926 cells (Antigny et al., 2012), a widely employed HUVECs-derived cell line. In addition, vascular development was not impaired in TRPC1-deficient mice, while endothelium-dependent hyperpolarization was exacerbated (Schmidt et al., 2010). Further studies are, therefore, required to confirm the pro-angiogenic role of TRPC1.

##### TRPC4

Unlike TRPC1, genetic deletion of TRPC4 via specific small interfering RNAs (siRNAs; siTRPC4, in this case) impaired spontaneous Ca^2+^ oscillations and tube formation in EA.hy926 cells (Antigny et al., 2012). Consistently, TRPC4 mediate VEGF-induced neovascularization in hypoxic retina (Song et al., 2015). Oxygen depletion increased TRPC4 expression in mouse retina, whereas the intravitreal infusion of a specific siTRPC4 impaired the subsequent process of retinal neovascularization (Song et al., 2015). Accordingly, genetic deletion of TRPC4 prevented VEGF-induced migration and tube formation by interfering with the ERK and Akt signaling pathways in human retinal microvascular endothelial cells (Song et al., 2015). Earlier work carried out on human pulmonary artery endothelial cells (PAECs) revealed that hypoxia leads to TRPC4 up-regulation through the AP-1 transcription factors and that TRPC4 was able to mediate SOCE by carrying the I_SOC_ (Fantozzi et al., 2003). Furthermore, TRPC4 could play a crucial role in oxidized low-density lipoprotein (oxLDL)-induced *in vitro* angiogenesis. OxLDL-induced proliferation, migration and tube formation in human coronary artery endothelial cells (HCAECs) were impaired by transfecting the cells with a specific siTRPC4 (Qin et al., 2016). TRPC4 induced angiogenesis by promoting VEGF release and inducing the nuclear translocation of the Ca^2+^-sensitive transcription factor, NF-κB (Qin et al., 2016). It has, therefore, been suggested that TRPC4 might provide an alternative molecular target to treat atherosclerotic neovascularization. Of note, TRPC4 could be preferentially recruited by EGF to the plasma membrane in proliferating endothelial cell clusters, which establish immature cell-to-cell contacts (Chen et al., 2016). TRPC4 is then retrieved from the plasma membrane upon formation of mature endothelial barriers and is relocated within junctional complexes, where it is barely available to physiological stimuli (Graziani et al., 2010). This phenotype-dependent subcellular localization of TRPC4 could further help understand why some reports identified this channel as a pore-forming subunit of endothelial I_SOC_ (Fantozzi et al., 2003; Sundivakkam et al., 2012) or I_CRAC_ (Freichel et al., 2001; Cioffi et al., 2005), while others failed (Abdullaev et al., 2008).

#### TRPC3 and TRPC6 in Angiogenesis

While the role played by TRPC1 and TRPC4 channels in VEGF-induced SOCE is controversial, there is no doubt that TRPC3 and TRPC6 may sustain VEGF-induced extracellular Ca^2+^ entry and angiogenesis in vascular endothelial cells (Figure 2). TRPC3 and TRPC6 are gated by the PLCγ1 metabolite, DAG, in a protein kinase C (PKC)-independent manner (Moccia et al., 2018b). An earlier report suggested that a heteromeric TRPC3/TRPC6 channel was involved in VEGF-induced Ca^2+^ influx in human microvascular endothelial cells (HMVECs) (Cheng et al., 2006). Subsequently, the same group demonstrated that VEGF-induced extracellular Ca^2+^ entry, proliferation and tube formation were impaired in HMVECs overexpressing a dominant negative TRPC6 construct (Hamdollah Zadeh et al., 2008). Similarly, TRPC3 and TRPC6 could sustain VEGF-induced extracellular Ca^2+^ entry in HUVECs. Pharmacological (by means of selective inhibitors) and genetic (by using selective siRNAs) manipulation demonstrated that TRPC3-mediated Na^+^ entry could switch the Na^+^/Ca^2+^ exchanger (NCX) in the reverse (Ca^2+^ entry) mode (Andrikopoulos et al., 2011a, 2017). The subsequent increase in [Ca^2+^]_i_ was sufficient to recruit PKC-α and engage the ERK signaling pathway, thereby promoting VEGF-induced proliferation and tube formation in HUVECs (Andrikopoulos et al., 2011a, 2017). TRPC6 may also sustain VEGF-induced Ca^2+^ entry in HUVECs (Ge et al., 2009). Likewise, genetic deletion of TRPC6 with a specific siRNA reduced VEGF-induced extracellular Ca^2+^ entry, proliferation and tube formation *in vitro* as well as angiogenesis *in vivo* (Ge et al., 2009). However, TRPC6 was not involved in the pro-angiogenic Ca^2+^ response to bFGF (Ge et al., 2009). Interestingly, a selective siTRPC3 was able to impair spontaneous Ca^2+^ oscillations and assembly into a bidimensional tubular network in EA.hy926 cells, while genetic deletion of TRPC6 was without consequence (Antigny et al., 2012). Additional investigations demonstrated that TRPC3 and TRPC6 may drive endothelial cell proliferation, motility and tube formation in response to pro-angiogenic cues other than VEGF. For instance, erythropoietin-induced intracellular Ca^2+^ signals and migration in EA.hy926 cells were impaired by inhibiting TRPC3-mediated Ca^2+^ influx with Pyr3 and a blocking antibody selectively targeting TRPC3 (Maltaneri et al., 2018). In addition, the Ca^2+^ response to erythropoietin was sensitive to pharmacological blockade of L-type voltage-gated Ca^2+^ channels (VGCCs) with diltiazem and amlodipine (Maltaneri et al., 2018). It should, however, be pointed out that CaV1 subunits, which mediate L-type Ca^2+^ currents, are yet to be identified in vascular endothelial cells (Moccia et al., 2012a) and that a recent report demonstrated that endothelial SOCE is also sensitive to a number of drugs targeting L-type VGCCs (Mergler et al., 2003). TRPC6, in turn, mediates (±)-11,12-EET- and thrombin-induced migration and tube formation in HUVECs (Ding et al., 2014) and human PAECs (Kini et al., 2010). Interestingly, thrombin-induced TRPC6-mediated extracellular Ca^2+^ entry is tightly regulated by the dual lipid-protein phosphatase, phosphatase and tensin homologue (PTEN). PTEN was found to physically interact with TRPC6 through its tail domain (residues 394-403), thereby promoting the membrane translocation of TRPC6 and Ca^2+^ influx upon exposure to thrombin (Kini et al., 2010). Conversely, TRPC6 can be activated by lysophosphatidylcholine, the most abundant lysophospholipid in oxLDL, to inhibit wound closure in cultured mouse aortic endothelial cells and carotid artery regeneration in a mouse model of hypercholesterolemia (Rosenbaum et al., 2015). Therefore, the role of TRPC6 in vascular remodeling could be tightly modulated by local microenvironment and be dampened under pro-atherosclerotic conditions.

#### TRPC5 in Angiogenesis

TRPC5 is also able to modulate angiogenesis in several vascular beds (Figure 2) (Smani et al., 2018). TRPC5 mediates extracellular Ca^2+^ entry downstream PLC activation, although the gating role of STIM proteins and ER Ca^2+^ store emptying remains to be fully elucidated (Zholos, 2014; Moccia, 2018). A number of chemical mediators may activate TRPC5 by acting on the extracellular side. These include, but are not limited to, NO, H^+^, Pb^2+^, lanthanides, reduced thioredoxin, and Ca^2+^, which may also act intracellularly (Zholos, 2014). Finally, TRPC5 is sensitive to membrane stretch and to cold temperatures (37°C to 25°C) (Zholos, 2014). Earlier work showed that TRPC5 may negatively affect cell migration in BAECs. According to these studies, TRPC6-mediated Ca^2+^ influx recruits myosin light chain kinase (MLCK) in an ERK- and NADPH oxidase-dependent manner, thereby inducing TRPC5 to translocate toward the plasma membrane and inhibit BAEC motility (Chaudhuri et al., 2008, 2017). Recently, however, TRPC5 was found to sustain intracellular Ca^2+^ oscillations and tube formation in EA.hy926 cells (Antigny et al., 2012). Furthermore, it has been shown that genetic deletion of TRPC5 with a selective siRNA impaired hypoxia-induced capillary sprouting and tube formation in primary mouse intestinal mesenteric vascular endothelial cells (Zhu et al., 2019). Consistently, hypoxia-induced retinal neovascularization was compromised in a mouse model deficient of TRPC5 (Zhu et al., 2019). Finally, genetic knockdown of TRPC5 prevented vascular revascularization in a mouse model of hindlimb ischemia at 14 days post-intervention. Intriguingly, vessel density and blood flow recovery were enhanced by over-expressing TRPC5 upon injection of an adeno-associated virus construct encoding for TRPC5 or by administrating riluzole, the first medication approved by the Food and Drug Administration to treat amyotrophic lateral sclerosis (Zhu et al., 2019). As widely reviewed in Bellingham (2011), riluzole may inhibit voltage-gated Na^+^ and Ca^2+^ channels, whereas it can stimulate Ca^2+^-dependent and voltage-independent two-pore K^+^ currents. However, recent studies revealed that riluzole may also activate TRPC5 with no requirement for PLC engagement (Richter et al., 2014a, b). In agreement with these observations, riluzole elicited non-selective cation currents and extracellular Ca^2+^ entry also in mouse mesenteric vascular endothelial cells (Zhu et al., 2019). TRPC5-mediated Ca^2+^ entry stimulated angiogenesis by promoting the nuclear translocation of NFAT isoform c3 (NFATc3) and angiopoietin 1 expression (Zhu et al., 2019). Similar to TRPC6, however, TRPC5 activation by oxidized lipids may prevent endothelial wound closure *in vitro* and regeneration of endothelial monolayers *in vivo* (Rosenbaum et al., 2015).

### The Role of TRPV Channels in Angiogenesis and Arteriogenesis

**FIGURE 3:**
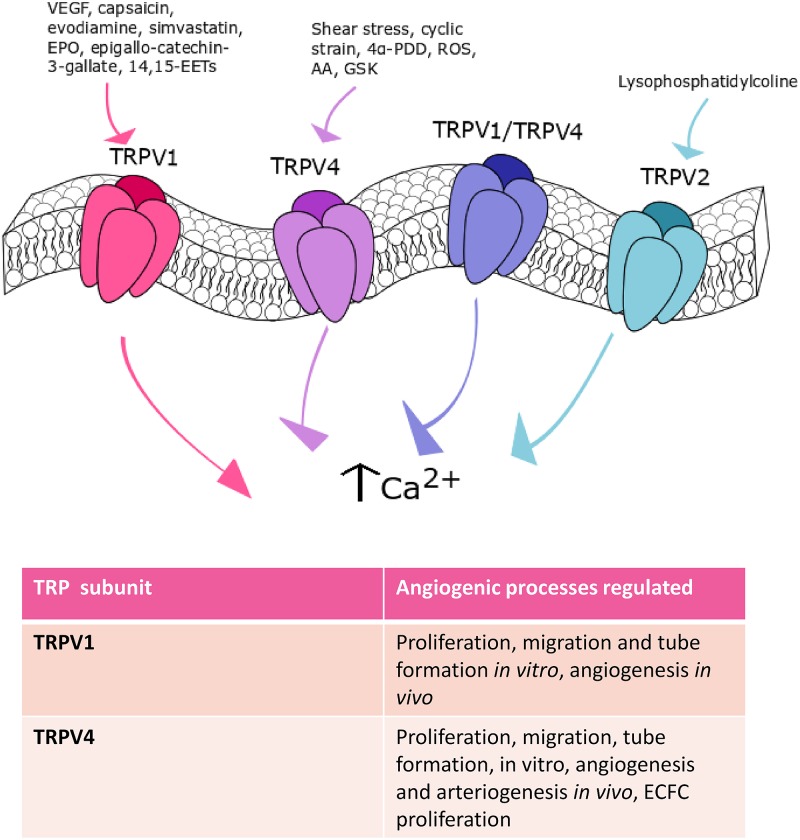
TRPV channels in angiogenesis. TRPV1, TRPV2, and TRPV4 are the main TRPV isoforms that mediate angiogenesis. TRPV1 is involved in angiogenesis in response to: VEGF, capsaicin, evodiamine, simvastatin, EPO, epigallocatechin-3-gallate, and 14,15-EETs. Shear stress-, cyclic strain-, 4α- PDD-, ROS-, AA- and GSK-mediated TRPV4 activation participate to angiogenesis. Finally, TRPV2 stimulation with lysophosphatidylcoline can play a role in this process.

The TRPV subfamily encompasses 6 isoforms, TRPV1-6. However, only the TRPV isoforms 1-4 (TRPV1-4) play clear roles in vascular function (Earley and Brayden, 2015), while TRPV5 and TRPV6 channels are mainly located in the luminal membrane of renal and intestinal epithelium (Gees et al., 2010). TRPV1-4 are non-selective cation channels, which are featured by a permeability ratio P_Ca_/P_Na_ between ∼1 and ∼15 (Gees et al., 2010). Conversely, TRPV5 and TRPV6 represent a separate group, are less similar (22–24% identity) to other TRP isoforms and are highly Ca^2+^-selective (Gees et al., 2010). TRPV1-4 channels serve as polymodal Ca^2+^-entry pathways being activated by multiple cues, such as temperature increases, mechanical stimuli and chemical agonists (Gees et al., 2010; Earley and Brayden, 2015). They are known to regulate a plethora of endothelial functions, including vascular tone and permeability, inflammation, and vascular remodeling (Earley and Brayden, 2015; Guerra et al., 2018; Smani et al., 2018; Moccia et al., 2019a; Thakore and Earley, 2019). Herein, we will focus on TRPV1 and TRPV4, which are the most important endothelial TRPV isoforms involved in angiogenesis and arteriogenesis (Figure 3). Furthermore, TRPV1 and TRPV4 channels may control vascular remodeling by promoting VSMC proliferation and migration under pathological conditions, such as pulmonary hypertension (Inoue et al., 2006; Randhawa and Jaggi, 2015).

#### TRPV1 in Angiogenesis

TRPV1, also known as the vanilloid receptor 1 (VR1), was the first mammalian TRPV family member to be discovered and the most extensively studied (Gees et al., 2010). TRPV1 is an archetypal polymodal channel as it is activated by distinct physical and chemical stimuli, such as noxious heat (>42°C) and extracellular protons, plants-derived products (e.g., capsaicin and gingerol), hydrogen sulfide (H_2_S), and tarantula spider-derived vanillotoxins (Bevan et al., 2014). Multiple evidences demonstrated that endothelial TRPV1 may stimulate angiogenesis (Smani et al., 2018; Thakore and Earley, 2019), although earlier work suggested that capsaicin, the most widely employed TRPV1 agonist, actually exerts an anti-angiogenic effect (Min et al., 2004). This study showed that capsaicin was able to inhibit VEGF-induced DNA synthesis, proliferation, migration and tube formation in HUVECs. In addition, capsaicin was found to impair VEGF-induced vascularization in Matrigel plugs *in vivo* (Min et al., 2004). This study, however, did not assess TRPV1 contribution in the anti-angiogenic effect of capsaicin. Furthermore, a subsequent investigation revealed that intraperitoneal administration of evodiamine, a TRPV1 agonist, induced angiogenesis in Matrigel plugs *in vivo* (Ching et al., 2011). TRPV1-mediated extracellular Ca^2+^ entry promoted PI3K/Akt/CaMKII signaling, which in turn led to: (1) TRPV1 phosphorylation, (2) physical interaction between eNOS and TRPV1; (3) eNOS phosphorylation and, ultimately, (4) NO release (Ching et al., 2011). Accordingly, evodiamine failed to induce angiogenesis when Matrigel plugs were implanted in eNOS- or TRPV1-deficient mice (Ching et al., 2011). Of note, it has long been known that NO induces endothelial cell proliferation, migration, and tube formation (Mancardi et al., 2011). Moreover, TRPV1 induced AMP-activated protein kinase (AMPK) phosphorylation, which favored the formation of a TRPV1-eNOS complex, thereby boosting NO release and angiogenesis both in Matrigel plugs and in a mouse model of hindlimb ischemia (Ching et al., 2012). In addition, TRPV1 mediates simvastatin-, erythropoietin-, epigallocatechin-3-gallate- and 14,15-EET-induced angiogenesis. Simvastatin, a 3-hydroxy-3-methylglutaryl-CoA reductase blocker that is employed to treat hypercholesterolaemia and cardiovascular disorders, stimulated *in vivo* angiogenesis by promoting NO release through the assembly of the TRPV1-Akt-CaMKII-AMPK-eNOS complex described above (Su et al., 2014b). The same signaling pathway is recruited to promote tube formation *in vitro* and vascular development *in vivo* by erythropoietin (Yu et al., 2017) and epigallocatechin-3-gallate, the main catechin present in the green tea (Guo et al., 2015). Finally, pharmacological (with capsazepine) and genetic (with a selective siRNA) inhibition of TRPV1 prevented 14,15-EET-induced Ca^2+^ influx, NO release, *in vitro* tube formation and *in vivo* angiogenesis in HMECs (Su et al., 2014a). A recent investigation demonstrated that TRPV1 may assemble into a heteromeric complex with TRPV4 in mouse retinal microvascular endothelial cells (RMECs) (O’Leary et al., 2019). The TRPV1/TRPV4 heteromeric channel did not promote VEGF-induced extracellular Ca^2+^ entry and sprouting angiogenesis, but it was *per se* able to mediate Ca^2+^ influx and drive RMEC proliferation, motility and capillary-like tube formation (O’Leary et al., 2019). While these studies hinted at extracellular Ca^2+^ entry as the main mechanism whereby TRPV1 induces angiogenesis, an alternative signaling mode has been described in EA.hy926 cells (Hofmann et al., 2014). Herein, Hofmann et al. (2014) demonstrated that TRPV1 mediated the uptake of the endogenous cannabinoid, anandamide, in a Ca^2+^-independent manner. The intracellular transport of anandamide, in turn, promoted EA.hy926 cell proliferation and tube formation (Hofmann et al., 2014), which is consistent with the emerging notion that TRP channels drive angiogenesis independently on their ability to conduct extracellular Ca^2+^ (Abdullaev et al., 2008). These studies strongly suggest that the pharmacological stimulation of TRPV1 represents a promising pathway to induce angiogenesis in the presence of cardiovascular risk factors (i.e., hypercholesterolaemia) or upon an ischemic insult.

##### Endothelial TRPV1 in Vascular Remodeling

Early work revealed that endothelial TRPV1 may also be responsible for vascular remodeling observed in rat model of arteriovenous fistula (AVF) created at the femoral artery and vein (Chen et al., 2010). The following increase in blood flow led to the shear stress-induced activation of TRPV1, which in turn caused venodilation, wall thickening and vascular remodeling in fistula veins. These effects were exacerbated by the concomitant up-regulation of endothelial TRPV1. It was demonstrated that TRPV1 recruited CaMKII to phosphorylate eNOS and induce NO-dependent MMP2 activation (Chen et al., 2010). The pharmacological blockade of TRPV1 prevented the increase in shear stress and vascular remodeling observed upon AVF formation by inhibiting the signaling pathways engaged by extracellular Ca^2+^ entry (Chen et al., 2010). These findings suggest that endothelial TRPV1 could be also activated by mechanical deformation of the plasma membrane, as previously demonstrated in retinal ganglion cells (Sappington et al., 2009) and in the mechanosensitive fibers of renal pelvis (Feng et al., 2008).

#### TRPV4 in Angiogenesis

Originally, TRPV4 was identified as a mechanosensor or osmosensor, activated by hypotonic osmotic stress and shear stress (Shibasaki, 2016). Subsequently, TRPV4 was found to be heat-sensitive, being activated by temperatures greater than 27 °C (Shibasaki, 2016). TRPV4 can be also gated by synthetic agonists, such as the phorbol ester 4α-phorbol 12,13-didecanoate (4α-PDD), by acidic pH and citrate and, finally, by endogenous agonists, such as the endocannabinoid anandamide, AA, and its hydrolytic products 5,6-EETs and 14,15-EETs (White et al., 2016). TRPV4 channels display higher selectivity to Ca^2+^ than Na^+^ ions and mediate outward rectifier ionic currents (White et al., 2016). Early work demonstrated that mechanical stimulation of TRPV4 by cyclic strain induced bovine capillary endothelial cells to realign perpendicular to the direction of the strain upon PI3K-dependent β1 integrin recruitment, stress fiber and focal adhesion rearrangement (Thodeti et al., 2009). Interestingly, the same group recently demonstrated that genetic silencing of TRPV4 increased the cell surface expression and phosphorylation at Y1175 of VEGFR2 in HMECs, thereby stimulating VEGF-induced migration (Kanugula et al., 2019). The emerging role played by TRPV4 in angiogenesis was further corroborated by Hatano et al. (2013), who found that TRPV4 activation by 4α-PDD induced proliferation in human brain capillary endothelial cells. In agreement with this observation, the intra-tail injection of 4α-PDD at days 1-4 in rats undergoing transient brain ischemia decreased infarct volume by ≈50% and significantly reduced neurological deficit by promoting brain angiogenesis and neurogenesis (Chen et al., 2018). TRPV4 activation was shown to induce eNOS phosphorylation, to stimulate VEGF levels in the ischemic area and to up-regulate VEGFR2 expression (Chen et al., 2018). TRPV4-mediated extracellular Ca^2+^ entry has been implicated also in neovascular diseases, such as PAH (Suresh et al., 2018) and cancer (see Section Remodeling of Endothelial TRP Channels Promote Aberrant Tumor Vascularization). Accordingly, preliminary work demonstrated that oxidative stress was able to dismantle endothelial permeability by stimulating the Src kinase Fyn to activate TRPV4 in human and mouse lung microvascular endothelial cells (Suresh et al., 2015). Recently, it has been demonstrated that the higher levels of cytosol- and mitochondria-derived ROS led to the constitutive opening of TRPV4, thereby increasing resting [Ca^2+^]_i_ and promoting aberrant endothelial cell proliferation and migration (Suresh et al., 2018). This observation strongly suggests that endothelial TRPV4 is involved in PAH pathogenesis.

#### TRPV4 in Arteriogenesis

As mentioned earlier, arteriogenesis refers to the remodeling of pre-existing arterio-arteriolar anastomoses to completely developed and functional arteries (Fischer et al., 2006). Earlier work provided the evidence that TRPV4 transcription was increased by the elevated shear stress driving collateral vessel growth after femoral artery ligature (FAL) in rats (Troidl et al., 2009). Furthermore, 4α-PDD, a TRPV4 agonist (see Paragraph TRPV4 in Angiogenesis), stimulated proliferation in porcine aortic endothelial cells *in vitro* and promoted collateral vessel growth and local tissue perfusion after FAL *in vivo* (Troidl et al., 2009). Of note, the infusion of 4α-PDD failed to stimulate arteriogenesis and rescue blood supply in a mouse model KO for TRPV4 (Troidl et al., 2009). Subsequently, the same group confirmed the role of TRPV4 in arteriogenesis also in a porcine model of hindlimb-ischemia (Troidl et al., 2010). In addition, a number of Ca^2+^-dependent transcription factors were found to mediate the pro-arteriogenic effect of TRPV4 in pigs, including NFAT, myocyte enhancer factor 2C and DREAM (Troidl et al., 2010). Finally, endothelial TRPV4 expression was also increased in cerebral collateral circulation after bilateral carotid artery ligature (BCL) (Schierling et al., 2011). Similar to FAL (Troidl et al., 2009), 4α-PDD treatment led to enhanced collateral growth *in vivo* by inducing endothelial cell proliferation (Schierling et al., 2011). These observations reinforce the tenet that the pharmacological stimulation of TRPV4 provides an alternative therapeutic strategy to restore blood perfusion in damaged or obstructed vessels.

#### TRPV1 and TRPV4 in VSMCs

TRPV1 and TRPV4 could induce vascular remodeling by also stimulating proliferation and migration in pulmonary artery VSMCs. Early work demonstrated that TRPV1 and TRPV4 were expressed, mediated extracellular Ca^2+^ entry and supported migration through cytoskeletal remodeling in these cells (Martin et al., 2012). Subsequently, it has been shown that chronic hypoxia increased TRPV1 and TRPV4 expression in pulmonary artery VSMCs, thereby increasing the Ca^2+^ response to mechanical stimulation, the rate of cell motility, and the nuclear translocation of NFATc4 (Parpaite et al., 2016). In agreement with these observations, TRPV4 protein was up-regulated in a rat model of PAH, which led to an increase in membrane swelling- and 4α-PDD-induced extracellular Ca^2+^ entry in VSMCs (Yang et al., 2012). Furthermore, TRPV4 overexpression caused a significant elevation in resting [Ca^2+^]_i_, which ultimately resulted in an increased myogenic tone. Of note, development of PAH, vascular remodeling and right ventricle hypertrophy induced by chronic hypoxia were prevented in a mouse model deficient of TRPV4 (Yang et al., 2012). These observations, therefore, hint at TRPV4 as a crucial player in both endothelial dysfunction (see Paragraph TRPV4 in Angiogenesis) and vascular remodeling in PAH. Nevertheless, a recent investigation confirmed that TRPV1 protein was up-regulated in pulmonary artery VSMCs harvested from subjects affected by idiopathic PAH (IPAH). Moreover, capsaicin- and membrane swelling-induced Ca^2+^ entry were enhanced and led to an increase in CREB phosphorylation, which was associated to the higher proliferation rate of IPAH VSMCs (Song et al., 2017). The role of TRPV4 in PAH remains to be confirmed in human samples.

### The Role of TRPM Channels in Angiogenesis

**FIGURE 4:**
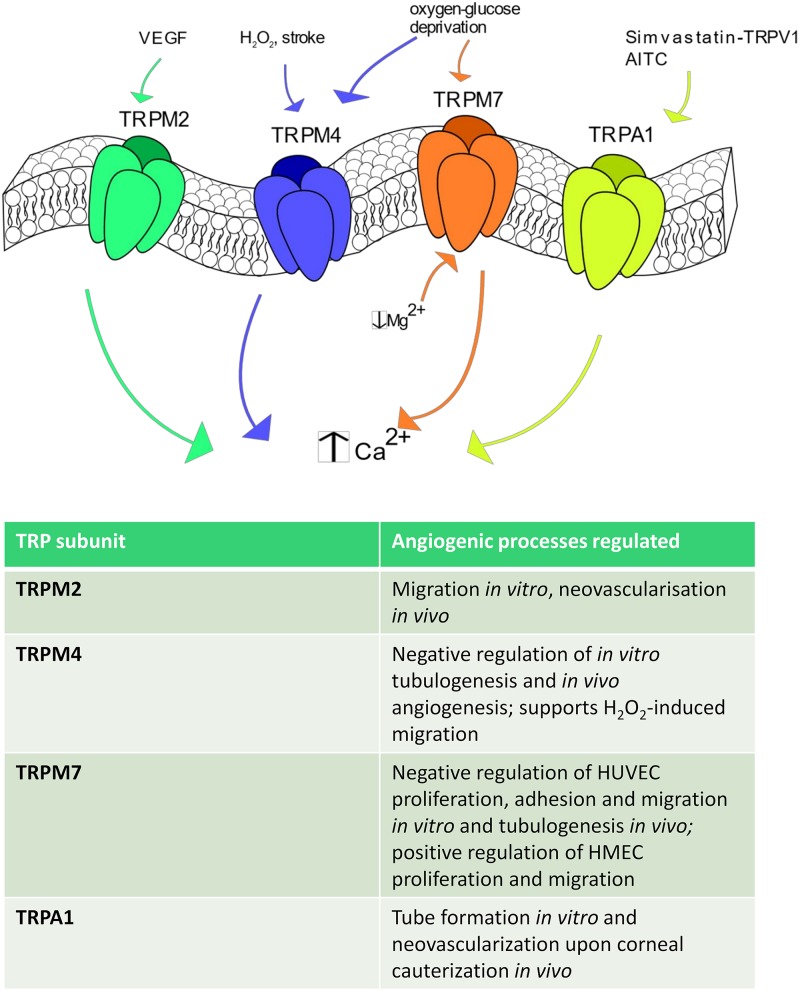
TRPM and TRPA1 channels in angiogenesis. Some TRPM isoforms are shown to be involved in angiogenesis. In particular, TRPM2 stimulates angiogenesis after VEGF stimulation, whereas TRPM4 is activated by H2O2, stroke and oxygen-glucose deprivation. TRPM7 regulates endothelial cell proliferation and tube formation being sensitive to a reduction in intracellular Mg2**^+^** concentration and to oxygen-glucose deprivation. Finally, TRPA1 promotes angiogenesis in response to AITC and upon simvastatin-dependent TRPV1 activation.

The TRPM subfamily consists of 8 members, TRPM1-8, named based upon the first member, i.e., TRPM1, that has been first identified as tumor suppressor in melanoma (Gees et al., 2010). TRPM channels may be classified in three separate groups based on their sequence homology: TRPM1/TRPM3, TRPM4/TRPM5; TRPM6 and TRPM7; TRPM2 and TRPM8, which bear scarce amino-acid sequence similarity (Ramsey et al., 2006). TRPM channels are featured by a variable permeability to Ca^2+^, which ranges from the Ca^2+^ impermeable channels TRPM4 and TRPM to the highly Ca^2+^ permeable TRPM3, TRPM6 and TRPM7 (Gees et al., 2010). Herein, we focus on TRPM2, TRPM4 and TRPM7 (Figure 4), which represent the sole TRPM members involved in angiogenesis (Smani et al., 2018).

#### TRPM2

TRPM2 is equally permeable to Na^+^ and Ca^2+^ (PCa/PNa ≈ 0.7–0.9) and is gated by the intracellular second messenger ADPr, which binds to its COOH-terminal NUDIX domain, H_2_O_2_ and compounds that generate reactive oxygen/nitrogen species (Faouzi and Penner, 2014; Thakore and Earley, 2019). A recent investigation revealed that VEGF induced Ca^2+^ influx through TRPM2 in a ROS-dependent manner in human pulmonary artery endothelial cells (Mittal et al., 2015). Furthermore, extracellular Ca^2+^ entry induced TRPM2 to assemble with c-Src and VE-cadherin at adherens junctions, thereby causing VE-cadherin internalization and stimulating endothelial cell migration *in vitro* (Mittal et al., 2015). Accordingly, VEGF failed to promote neovascularization of Matrigel plugs in TRPM2 KO mice as well as blood flow recovery was severely impaired in a TRPM2 KO mice model of hindlimb ischemia (Mittal et al., 2015). Finally, microvascular density and restoration of post-ischemic blood flow were severely compromised in a TRPM2 KO mouse model (Mittal et al., 2015). For all these studies, the TRPM2 KO mice were provided by GlaxoSmithKline and were devoid of side effects (Mittal et al., 2015).

#### TRPM4

As mentioned earlier, TRPM4, as well as its companion TRPM5, represents a peculiar TRP channel as it is permeable only to monovalent cation, although it is gated by an increase in [Ca^2+^]_i_ (Gees et al., 2010). It has been shown that oxygen-glucose deprivation was able to increase TRPM4 expression and impair *in vitro* tubulogenesis in HUVECs. Nevertheless, pharmacological blockade of TRPM4 with 9-phenanthrol restored their pro-angiogenic behavior (Loh et al., 2014). Notably, cerebral stroke induced in rats by permanent occlusion of the middle cerebral artery resulted in endothelial TRPM4 up-regulation and remarkable loss of endothelial integrity within the penumbra region (Loh et al., 2014). However, local injection of a siTRPM4 prevented vascular dysfunction and increased capillary density, which hints at TRPM4 as a negative modulator of angiogenesis (Loh et al., 2014). Accordingly, the brain infarct volume and recovery of motor function were enhanced by genetic deletion of TRPM4, although the protective effect of TRPM4 silencing on motor function was only transient and ceased at day 5 post-intervention (Loh et al., 2014). It should, however, be pointed out that TRPM4 was also found to enhance H_2_O_2_-induced HUVEC depolarization and migration (Sarmiento et al., 2015). Therefore, the role played by TRPM4 in angiogenesis could depend on the surrounding context and/or the endothelial cell type, as noticed for other endothelial TRP isoforms.

#### TRPM7

TRPM7 provides a constitutively open pathway for Mg^2+^, Zn^2+^, and Ca^2+^, and is regulated by multiple signals, including PIP_2_ hydrolysis, decrease in free cytosolic Mg^2+^ levels and cell volume changes (Fleig and Chubanov, 2014). Earlier work confirmed that TRPM7 was gated by a drop in intracellular Mg^2+^ levels in HUVECs and revealed that genetic deletion with a specific siRNA increased HUVEC proliferation by enhancing ERK phosphorylation, eNOS expression and NO release (Inoue and Xiong, 2009). Conversely, genetic deletion of TRPM7 stimulated HUVEC adhesion to the substrate, migration in the wound healing assay and tubulogenic activity in Matrigel (Zeng et al., 2015). In agreement with the previous finding, TRPM7 silencing promoted ERK phosphorylation (Zeng et al., 2015). A subsequent investigation reported that a selective siTRPM7 reduced HMEC proliferation by inducing cell cycle arrest in the G0/G1 and G1 phases and impaired HMEC migration, while it did not affect *in vitro* tube formation (Baldoli and Maier, 2012). A more recent investigation disclosed that TRPM7 negatively modulates migration and tube formation in brain microvascular endothelial cells both after oxygen-glucose deprivation *in vitro* and after cerebral stroke *in vivo* (Yang et al., 2018). This study further revealed that TRPM7 was down-regulated by the adipose-derived stem cells (ADSCs)-derived exosomal microRNA-181b-5p (181-bExos) (Yang et al., 2018). Notably, TRPM7 expression was decreased, while endothelial cell migration and tube formation were enhanced, by the ADSCs-derived 181-bExos (Yang et al., 2018). Overall, it appears that endothelial TRPM channels might not provide the best means to stimulate therapeutic angiogenesis. Accordingly, while the pro-angiogenic role of TRPM2 remains to be confirmed also by future investigations, TRPM4 and TRPM7 emerge as negative modulator of the angiogenic process. Notably, the observation that their expression levels are increased under hypoxic conditions strongly suggest that TRPM4 and TRPM7 play a crucial role in limiting the revascularization process of ischemic tissues.

### The Role of TRPA1 in Angiogenesis

TRPA1 displays higher permeability to Ca^2+^ than Na^+^ (PCa/PNa = 7.9), presents a significant fractional Ca^2+^ current, and is activated by dietary compounds, such as allicin and AITC, mechanical deformation, and thermal stimuli (Thakore and Earley, 2019). Interestingly, the pro-angiogenic effect of simvastatin was prevented also in TRPA1 null mice, in which the simvastatin-induced formation of the TRPV1-Akt-CaMKII-AMPK-eNOS complex was also compromised (Su et al., 2014b). This finding led to the hypothesis that TRPA1 is the final effector of simvastatin-induced TRPV1 signaling. Conversely, the direct stimulation of TRPA1 with AITC did not induced HUVEC proliferation and migration neither was involved in the pro-angiogenic response to VEGF (Usui-Kusumoto et al., 2019). However, this investigation also reported that TRPA1 activation by VEGF on surrounding cells promoted tube formation when HUVEC were grown on a feeder monolayer of fibroblasts and to promote stromal neovascularization upon corneal cauterization (Usui-Kusumoto et al., 2019).

## TRP Channels Regulate Vasculogenesis

EPCs are released in peripheral circulation throughout postnatal life to replace damaged/senescent endothelial cells, to rescue the vascular network and restore local blood supply upon an ischemic insult, or to support tumor growth and vascularization in solid malignancies (Yoder, 2012; Moccia and Guerra, 2016; Poletto et al., 2018). The term EPC actually encompasses two distinct cellular populations that belong to diverse lineages and exploit different mechanisms to support vasculogenesis. These, respectively, include myeloid angiogenic cells (MACs) and endothelial colony forming cells (ECFCs) (Medina et al., 2017). As discussed in a recent Consensus Statement on Nomenclature, MACs are myeloid cells (Medina et al., 2017), which are mobilized from bone marrow and support neovascularization by liberating paracrine signals that stimulate local angiogenesis and/or recruit ECFCs to the neovessel site. Conversely, ECFCs are truly endothelial precursors that are mobilized from vascular niches, sustain angiogenesis in a paracrine manner and provide the building blocks for neovessel formation. As illustrated below, TRP channels may regulate proliferation, tubulogenesis and neovessel formation in both human ECFCs and rodent MACs (Moccia and Guerra, 2016).

### The Role of TRPC1 and TRPC3 in Vasculogenesis

A thorough transcriptomic analysis revealed that peripheral blood-derived human ECFCs (PB-ECFCs) expressed only TRPC1 and TRPC4, while umbilical cord blood-derived ECFCs were also endowed with TRPC3 (Sanchez-Hernandez et al., 2010). Genetic silencing by means of specific siRNAs subsequently disclosed that TRPC1 interacts with STIM1 and Orai1 to generate SOCE in ECFCs (Li J. et al., 2011; Lodola et al., 2012). It is not clear whether Orai1 and TRPC1 form separate Ca^2+^-permeable channels, each of them being gated by STIM1, or whether they assemble into a supermolecular complex, which possibly involves TRPC4. Nevertheless, depletion of the ER Ca^2+^ pool failed to induce a detectable store-operated Ca^2+^ current in ECFCs (Li J. et al., 2011; Moccia et al., 2019b), which suggests that Orai1 and TRPC1 tightly interact to mediate an I_CRAC_-like conductance, as described in mouse PAECs (Cioffi et al., 2012). SOCE may be recruited, respectively, by stromal derived factor-1α (SDF-1α) to promote ECFC migration *in vitro* and neovessel formation *in vivo* (Zuccolo et al., 2018) and by VEGF to stimulate ECFC proliferation and tube formation (Dragoni et al., 2011, 2015b). Of note, SOCE engages the ERK 1/2 and PI3K/Akt signaling pathways to drive SDF-1α-induced ECFC motility (Zuccolo et al., 2018), whereas the pro-angiogenic response to VEGF involves the Ca^2+^-sensitive transcription factor, NF-κB (Dragoni et al., 2011). Similar to human ECFCs, STIM1, TRPC1 and Orai1 interact to mediate SOCE and support *in vitro* angiogenesis (proliferation, motility and the formation) in rodent MACs (Kuang et al., 2010, 2012; Wang et al., 2015). Furthermore, genetic knockdown of TRPC1 prevented neovessel formation in Matrigel plugs *in vivo* by impairing CaM-dependent eNOS activation (Du et al., 2018).

TRPC3, in turn, is selectively expressed in UCB-ECFCs (Sanchez-Hernandez et al., 2010), where it is physiologically gated by DAG and triggers VEGF-induced intracellular Ca^2+^ oscillations (Dragoni et al., 2013). Genetic (with specific siRNAs) and pharmacological (with Pyr3) manipulation revealed that TRPC3-mediated extracellular Ca^2+^ entry triggers the dynamic interplay between InsP_3_Rs and SOCE which shapes the spiking Ca^2+^ response to VEGF, thereby promoting UCB-ECFC proliferation (Dragoni et al., 2013). It has been suggested that TRPC3 involvement is responsible for the higher frequency of VEGF-induced intracellular Ca^2+^ oscillations in UCB-ECFCs, which is associated to their higher proliferative potential (Moccia et al., 2018a). This observation led to the hypothesis that the exogenous insertion of TRPC3 might rejuvenate the reparative phenotype of senescent/aging UCB-ECFCs, thereby improving the efficacy of autologous cell-based therapy in ischemic patients (Moccia et al., 2018a).

### The Role of TRPV1 and TRPV4 in Vasculogenesis

A series of independent investigations reported that, besides TRPC1, TRPC3 and TRPC4, human ECFCs also express TRPM7 (Baldoli and Maier, 2012), TRPV1 (Hofmann et al., 2014), and TRPV4 (Dragoni et al., 2015a). Genetic silencing of TRPM7 with a specific siRNA did not affect ECFCs’ proliferation rate (Baldoli and Maier, 2012), while selective stimulation of TRPV4 with GSK only exerted a weak effect on PB-ECFC growth (Dragoni et al., 2015a). Nevertheless, TRPV4-mediated extracellular Ca^2+^ entry supported AA-evoked intracellular Ca^2+^ signals, NO release and PB-ECFC proliferation (Dragoni et al., 2015a). This feature could become therapeutically relevant as AA is massively released in circulation during myocardial ischemia and upon brain damage (Moccia et al., 2018a). Unlike TRPV4, TRPV1 activation is *per se* sufficient to induce ECFC proliferation. An earlier investigation confirmed that TRPV1 drove UCB-ECFC proliferation by mediating anandamide uptake in a Ca^2+^-independent manner, as also reported in EA.hy926 cells (Hofmann et al., 2014). More recently, however, Lodola et al. (2019) revealed that optical excitation of PB-ECFCs with visible light (520 nm) induced TRPV1-mediated membrane depolarization when the cells were plated on the photo-sensitive polymer Poly(3-hexyl-thiophene) (P3HT). Visible light stimulation was able to promote TRPV1 opening by increasing ROS levels and local temperature within the cleft between the photo-sensitive substrate and cell surface, although a contribution of local pH was not ruled out. Notably, optical excitation of TRPV1 promoted PB-ECFC proliferation and tube formation by inducing the nuclear translocation of p65 NF-κB (Lodola et al., 2019). The pro-angiogenic effect of TRPV1 activation was prevented by buffering intracellular Ca^2+^ levels with BAPTA, thereby suggesting that Ca^2+^ influx through TRPV1 was necessary to induce angiogenesis (Lodola et al., 2019). It has, therefore, been suggested that optoceutical stimulation of TRPV1-mediated intracellular Ca^2+^ signals could represent an alternative strategy to improve the therapeutic outcome of regenerative medicine of ischemic disorders.

## Remodeling of Endothelial TRP Channels Promote Aberrant Tumor Vascularization

Recent pieces of evidence suggested that dysregulation of the endothelial Ca^2+^ machinery is crucial to support neovascularization and resistance to anti-angiogenic and chemotherapeutic drugs in a growing number of malignancies (Ma et al., 2014; Moccia, 2018; Scarpellino et al., 2019). Herein, we focus on the mechanisms whereby aberrant expression and/or activity of endothelial TRP channels impact on tumor vascularization.

### TRPV4 in Breast Cancer

TRPV4 has been the first endothelial TRP channel to be clearly associated to malignant angiogenesis. Accordingly, TRPV4 expression was remarkably enhanced in breast tumor-derived endothelial cells (B-TECs) and TRPV4 activation with AA or 4αPDD induced migration in B-TECs, but not control human cardiac microvascular endothelial cells (Pla et al., 2012) Conversely, AA-induced BTEC migration was prevented upon cell transfection with a short hairpin RNA selectively targeting TRPV4 (shTRPV4). Notably, AA-induced intracellular Ca^2+^ entry was larger in migrating, rather than non-migrating, B-TECs, and the cell surface expression of TRPV4 was significantly increased by a brief (10 min) pre-incubation with AA itself (Pla et al., 2012). Furthermore, earlier work demonstrated that AA was able to promote extracellular Ca^2+^ entry in B-TECs during the early steps of the tubulogenic process, but not within an established capillary-like network (Fiorio Pla et al., 2008). These data, therefore, strongly indicate that the up-regulation of endothelial TRPV4 channels plays a key role during the early phases of angiogenesis in breast cancer (BC).

### TRPV4 in Prostate Adenocarcinoma and Lewis Lung Carcinoma

Conversely, TRPV4 was down-regulated in prostate adenocarcinoma-derived endothelial cells (A-TECs), which displayed enhanced motility due to their lower mechanosensitivity toward extracellular matrix (ECM) stiffness. This, in turn, resulted in excessive tumor growth and vascularization in a mouse model deficient of TRPV4 and subcutaneously injected with Lewis Lung Carcinoma (LLC) cells (Adapala et al., 2016). Notably, tumor vasculature in TRPV4 KO mice presented a greater percentage of hyper-permeable, pericyte-free and dilated microvessels (Adapala et al., 2016), which are known to temper the therapeutic outcome of anticancer treatments (Moccia, 2018). A subsequent report disclosed that TRPV4 down-regulation caused a significant reduction in VE-cadherin expression at cell-to-cell contacts, which further contributed to increase vascular leakage (Cappelli et al., 2019). Overexpression or pharmacological stimulation of TRPV4 with GSK were, however, sufficient to normalize aberrant capillary-like tubules *in vitro* by restoring their mechanosensitivity toward ECM stiffness through the blockade of basal Rho activity (Thoppil et al., 2015; Adapala et al., 2016). Moreover, the daily intraperitoneal delivery of GSK improved pericyte coverage in TRPV4 KO mice xenografted with LLC cells and favored cisplatin-induced tumor shrinkage (Adapala et al., 2016). In addition, GSK-induced TRPV4-mediated extracellular Ca^2+^ influx inhibited the ERK 1/2 pathway, thereby decreasing A-TEC proliferation *in vitro* and reducing LLC growth *in vivo* (Thoppil et al., 2015). These findings, therefore, suggest that, depending on the tumor type, TRPV4 may be inhibited or activated to interfere with malignant vascularization.

### TRPVA1, TRPV2, and TRPC3 in Prostate Cancer

A recent investigation showed that endothelial TRP channels are remodeled also in prostate cancer (PCa). Bernardini et al. (2019) demonstrated that TRPA1, TRPV2 and TRPC3 were up-regulated in three different endothelial cell lines established from PCa patients and in the endothelial cells selectively lining tumor vessels *in vivo*. Pharmacological (with selective agonists) and genetic (with specific siRNAs) manipulation further revealed that: (1) TRPA1 supported PCa-derived endothelial cell migration; (2) TRPC3 promoted PCa-derived endothelial cell chemoattraction toward tumor microenvironment; and (3) TRPV2 induced capillary-like formation *in vitro* and vascular development in a mouse model of postnatal retina *in vivo* (Bernardini et al., 2019). The pro-angiogenic effect of TRP channels on PCa-derived endothelial cells was associated to a robust increase in intracellular Ca^2+^ activity as compared to non-tumor endothelium (Bernardini et al., 2019). Therefore, the pharmacological blockade of a number of selective TRP isoforms could represent a promising treatment for PCa patients. Conversely, endothelial TRPM8 down-regulation was found to regulate angiogenesis independently on Ca^2+^ signaling. Accordingly, TRPM8 was remarkably down-regulated in B-TECs as compared to HMECs and HUVECs, where it was mainly located within the ER (Genova et al., 2017). Genova et al. (2017) showed that TRPM8 inhibited migration and tube formation in normal endothelial cells by trapping Rap1 intracellularly, thereby preventing its relocation toward the plasma membrane, which is required to activate β1-integrin signaling. As a consequence, TRPM8 down-regulation in B-TECs is likely to accelerate vascular growth, thereby suggesting that TRPM8 activation (e.g., by icilin or menthol) could represent an efficient strategy to treat breast cancer.

### TRPC1 in Tumor-Derived ECFCs

TRPC1 may be heavily remodeled in tumor-derived ECFCs. For instance, TRPC1 and SOCE were up-regulated in renal cellular carcinoma (RCC)- (Lodola et al., 2012) and primary myelofibrosis (PMF)-derived ECFCs (Dragoni et al., 2014), but not in BC- and infantile hemangioma (IH)-derived ECFCs (Zuccolo et al., 2016; Lodola et al., 2017). Notably, pharmacological blockade of SOCE with BTP2 impaired proliferation and/or tube formation in RCC-, BC- and IH-derived ECFCs (Lodola et al., 2012, 2017), but not in PMF-derived ECFCs (Dragoni et al., 2014). As VEGF failed to induce pro-angiogenic Ca^2+^ oscillations in tumor-derived ECFCs (Lodola et al., 2012, 2017; Dragoni et al., 2015b; Zuccolo et al., 2016), a feature which might underpin the primary and secondary resistance to anti-VEGF drugs, TRPC1 (and SOCE) inhibition could represent a promising strategy to interfere with tumor vascularization (Moccia, 2018).

## Conclusion

Endothelial TRP channels serve a crucial role in vascular remodeling by regulating angiogenesis, arteriogenesis and vasculogenesis. The remarkable heterogeneity in their gating mechanisms, which can be further increased by the propensity of some isoforms to assemble into heteromeric complexes, render TRP channels the most versatile Ca^2+^ entry pathway in vascular endothelial cells, ECFCs and MACs. For instance, TRPC3 and TRPC6 mediate the pro-angiogenic response to VEGF, whereas TRPV4 senses the increase in laminar shear stress upon vascular occlusion, thereby promoting arteriogenesis. Nevertheless, endothelial TRP channels may also dampen angiogenesis, as reported for TRPM4 in HUVECs and TRPM7 in brain microvascular endothelial cells. Of course, a number of issues are yet to be clarified in order to fully appreciate and exploit the therapeutic potential of endothelial TRP channels. For instance, it is still unclear whether and how TRPC1 is recruited by VEGF in vascular endothelial cells and how it interacts with STIM1 and Orai1 to mediate VEGF-induced intracellular Ca^2+^ oscillations in ECFCs. Furthermore, while the role of endothelial TRP channels has been mainly investigated *in vitro*, the measurement of TRP channels-mediated endothelial Ca^2+^ signals *in vivo* remains a challenging issue. Recent investigations exploited a zebrafish model engineered to selectively express an endothelial genetic Ca^2+^ indicator (Yokota et al., 2015; Savage et al., 2019). Likely, these observations will pave the way for future studies aiming at assess the role of endothelial TRP channels *in vivo*. The sensitivity of endothelial TRPV channels to a moderate to large increase in local temperature also represents a puzzling issue (Watanabe et al., 2002; Mergler et al., 2010; Lodola et al., 2019). Accordingly, heat therapy induced a robust angiogenic switch in the myocardium of both healthy (Gong et al., 2006) and hypertensive (Ihori et al., 2016), whereas it rescued the capillary network and attenuated myocardial damage in a rat model of AMI (Sobajima et al., 2011). The contribution of thermosensitive TRPV1 and TRPV4 channels to heat-induced neovascularization requires to be evaluated. This hypothesis is corroborate by the recent evidence according to which TRPV1 may integrate local heat and ROS production to stimulate ECFC proliferation and tube formation (Lodola et al., 2019). An additional facet of endothelial TRP signaling that deserves further attention is its involvement in the abnormal vascularization that features multiple diseases, such as cancer, ischemic disorders and retinal degeneration. While remodeling of endothelial TRP channels has been largely appreciated, it is still unclear whether and which endothelial TRP isoforms are dysregulated, e.g., in coronary microcirculation of individuals suffering from AMI, atherosclerosis or hypertension. Exploring this issue, although technically challenging due to the difficulty of harvesting human endothelial samples, will permit to explore the therapeutic potential of endothelial TRP channels. For instance, pre-clinical trials already showed that stimulating TRPC5 with riluzole could represent a promising strategy to treat PAD, while the intra-myocardial/coronary injection of P3HT nanoparticles coupled to optical pacing of the heart could stimulate circulating ECFCs to rescue coronary circulation upon TRPV1 activation. Conversely, TRPV4 could be inhibited or stimulated to interfere with or normalize tumor vasculature in, respectively, BC and LLC.

## Author Contributions

FM and SN drafted and finally edited the manuscript. PF, RB-R, and GG significantly contributed to revise the manuscript. All the authors read and approved the final version of the manuscript.

## Conflict of Interest

The authors declare that the research was conducted in the absence of any commercial or financial relationships that could be construed as a potential conflict of interest.
